# Facteurs associés au risque de troubles du comportement alimentaire chez les étudiants en médecine de Casablanca, Maroc

**DOI:** 10.11604/pamj.2021.39.270.19976

**Published:** 2021-08-25

**Authors:** Nadia Attouche, Soukaina Hafdi, Rkia Somali, Omar Battas, Mohamed Agoub

**Affiliations:** 1 Laboratoire des Neurosciences Cliniques et Santé Mentale, Centre Psychiatrique Universitaire de Casablanca, Casablanca, Maroc

**Keywords:** Troubles du comportement alimentaire, étudiants en médecine, prévalence, facteurs de risque, Eating disorders, medical students, prevalence, risk factors

## Abstract

**Introduction:**

les troubles du comportement alimentaire (TCA) sont particulièrement fréquents chez les jeunes adultes, y compris les étudiants. Les objectifs de cette étude sont de déterminer la prévalence d'éventuels troubles du comportement alimentaire chez les étudiants en médecine de Casablanca et d´évaluer le risque de TCA selon les facteurs associés, notamment les facteurs socioéconomiques, cliniques, l´anxiété et la dépression.

**Méthodes:**

nous avons mené une étude transversale descriptive et analytique au niveau du Centre Hospiatlier Universiatire Ibn Rochd de Casablanca et à la faculté de médecine et de pharmacie de Casablanca Maroc, durant l´année universitaire 2016-2017. L´enquête a porté sur un échantillon de 506 étudiants. Nous avons utilisé le questionnaire SCOFF-F (Sick, Control, One stone, Fat, Food; version française), un outil validé pour le dépistage des troubles du comportement alimentaire et l´échelle HAD (Hospital anxiety and depression) pour dépister les troubles anxieux et dépressifs.

**Résultats:**

le questionnaire SCOFF a détecté 127 personnes (soit 25,09%) sur 506 susceptibles de souffrir de troubles du comportement alimentaire. L´âge, le niveau d´études, l´utilisation des moyens de contrôle du poids, l´anxiété et la dépression sont des facteurs associés de façon significative au risque de présenter un trouble du comportement alimentaire chez les étudiants.

**Conclusion:**

nos résultats sont en accord avec les données de la littérature concernant la fréquence des troubles du comportement alimentaire en population étudiante et plus spécifiquement les étudiants en médecine. Mener d´autres études sur une population plus variée pourraient également être envisagées afin d´avoir une vision plus globale de la situation au Maroc.

## Abstract (en)

**Introduction:**

eating disorders (EDs) are particularly common among young adults, including students. The purpose of this study is to determine eating disorder prevalence among medical students in Casablanca and to assess the risk of developing EDs on the bases of associated factors including socioeconomic factors, clinical features, anxiety and depression.

**Methods:**

we conducted a cross-sectional, descriptive and analytical study at the Ibn Rochd University Hospital and at the Faculty of Medicine and Pharmacy of Casablanca, Morocco, during the academic year 2016-2017. The survey involved a sample of 506 students. We used the SCOFF-F (Sick, Control, One Stone, Fat, Food, French version) questionnaire, a validated tool for eating disorders and the hospital anxiety and depression scale (HAD) for anxiety and depression.

**Results:**

the SCOFF questionnaire found that 127 people out of 506 (25.09%) were likely to suffer from eating disorders. Age, educational level, weight-management tools, anxiety and depression were significantly associated with the risk of developing eating disorders among medical students.

**Conclusion:**

our results are in agreement with data from the literature concerning eating disorder rates in the student population and, more specifically, among medical students. Further, studies of a more heterogeneous population are needed to have a more global vision of the situation in Morocco.

## Introduction

Les troubles du comportement alimentaire (TCA) désignent un groupe d'affections caractérisées par des habitudes alimentaires anormales. Ils impliquent soit un apport alimentaire insuffisant, soit un excès d´alimentation qui nuit à la santé physique et émotionnelle d´un individu. Le Bing *eating disorder*, la boulimie et l'anorexie sont considérées comme les formes les plus courantes des troubles du comportement alimentaire [1]. Des troubles infra-cliniques du comportement alimentaire surviennent de plus en plus souvent chez les enfants et les adolescents et peuvent avoir un impact considérable sur le développement psychosocial [2-4]. Les troubles de l'alimentation sont particulièrement fréquents chez les jeunes adultes, y compris les étudiants [5].

L'absence de signes prodromiques et l'absence de critères de diagnostic pendant les premiers stades du trouble entraînent souvent un diagnostic tardif. Les échelles de dépistage peuvent permettre un diagnostic et une intervention nutritionnelle précoces. L'une de ces échelles est le questionnaire SCOFF-F *(Sick, Control, One stone, Fat, Food)*, un test facilitant le dépistage et le diagnostic des troubles du comportement alimentaire chez les étudiants universitaires [6, 7].

Le comportement alimentaire est influencé par de nombreux facteurs biologiques, psychologiques, familiaux, socioculturels et divers facteurs multidimensionnels [8]. Les TCA sont d´origine multifactorielle et sont imputables à une association de facteurs de vulnérabilité biologique, sociale et psychologique [9].

Des études ont suggéré que les étudiants en médecine peuvent être considérés comme un groupe à haut risque de développer des problèmes de santé mentale, y compris les troubles du comportement alimentaire en raison du stress lié aux études, de la charge de travail extrêmement élevée, du besoin d'apprentissage continu et de l'exposition aux maladies et à la mort pendant la formation médicale [10-12]. Être un jeune adulte augmente également le risque de développer un trouble du comportement alimentaire en raison de la nature transitoire de cette phase de la vie en termes de relations, de concept de soi et d'objectifs pour l'avenir [13]. La stigmatisation associée aux troubles de santé mentale et du comportement alimentaire peut conduire au déni, à l'automédication, au diagnostic et aux traitements retardés, entraînant une présentation plus grave et persistante de ces troubles avec notamment des conséquences sur le fonctionnement global des étudiants [14].

En tenant compte des données précédentes et les rapports émergents sur l'importance d'explorer le fardeau des troubles du comportement alimentaire en population étudiante, notre étude vise à déterminer la prévalence d'éventuels troubles du comportement alimentaire chez les étudiants en médecine de Casablanca et d´évaluer le risque de TCA selon les facteurs associés, notamment les facteurs socioéconomiques, cliniques, l´anxiété et la dépression.

## Méthodes

**Type de l´étude:** étude transversale à visée descriptive et analytique, réalisée au niveau du CHU Ibn Rochd de Casablanca et à la faculté de médecine et de pharmacie de Casablanca Maroc, durant l´année universitaire 2016-2017.

**Population cible:** les étudiants en médecine de la première année à la sixième année.

**Échantillonnage:** cette étude transversale a été menée auprès de 506 étudiants. Un échantillonnage aléatoire simple a été utilisé. C´est une procédure de sélection aléatoire qui garantit que tous les membres de la population de la base de l´enquête ont la même probabilité de faire partie de la taille d'échantillon requise parmi 4398 étudiants.

**Déroulement de l´enquête et recueil des données:** pour les étudiants de 3^e^, 4^e^, 5^e^et 6^e^ année, et avec l'accord préalable des chefs de services, la distribution des questionnaires a été faite dans leurs salles de topos afin de leur expliquer l'intérêt et le but de l'étude. Ensuite, les étudiants, ayant accepté de participer à l'enquête, ont reçu les questionnaires à remplir, tout en respectant leur anonymat. Ces questionnaires, une fois remplis, ont été remis aux secrétaires de chaque service ou ramassé directement par les enquêteurs. Pour les étudiants de la 1^e^ et la 2^e^ la passation des questionnaires a été faite à la salle de lecture et au hall des amphithéâtres au sein de la faculté de Médecine et de Pharmacie de Casablanca.

**Critères d´inclusion:** le choix s´est porté sur les étudiants de la faculté de médecine et de pharmacie de Casablanca de la 1^e^, 2^e^, 3^e^, 4^e^, 5^e^et 6^e^ année.

**Critères d´exclusion:** les étudiants de la 7^e^ et 8^e^ année sont exclus en raison du stage interné dans des hôpitaux en périphérie. Les étudiants qui ont refusé de remplir le questionnaire ou qui étaient absents au moment de la distribution des questionnaires.

### Les questionnaires mal ou incomplètement remplis

**Instruments de mesure:** concernant le recueil des données, nous avons fait passer un auto-questionnaire anonyme:

***Données sociodémographiques et économiques:*** ces données incluent l´âge, le sexe, le statut matrimonial, le nombre d´enfants, le niveau socio-économique ainsi que le niveau d´étude.

***Données cliniques:*** elles incluent les antécédents médico-chirurgicaux et addictologiques, ainsi que le poids et la taille du sujet permettant de calculer son indice de masse corporelle (IMC). Nous avons également exploré les moyens de contrôle du poids éventuellement utilisés par les sujets.

***Questionnaire SCOFF:*** le questionnaire SCOFF-F *(Sick, Control, One stone, Fat, Food;* version française) est un outil validé pour le dépistage des troubles du comportement alimentaire [15]. Il est constitué de 5 questions dichotomiques (Oui/Non); répondre positivement à 2 questions ou plus indique une probabilité de présence d´un trouble du comportement alimentaire. Le SCOFF a été validé sur plusieurs populations et dans différentes langues [16]. Il est considéré comme un outil de dépistage des TCA, simple et efficace, particulièrement utilisé dans les populations estudiantines [17, 18].

***HAD:*** l´échelle HAD (Hospital anxiety and depression) est un instrument qui permet de dépister les troubles anxieux et dépressifs. Elle comporte 14 items cotés de 0 à 3. Sept questions se rapportent à l´anxiété (total A) et sept autres à la dimension dépressive (total D), permettant ainsi l´obtention de deux scores (note maximale de chaque score = 21).

**Consentement et aspect éthique:** pour chaque étudiant enquêté nous avons expliqué la définition des TCA, le but de l´étude. Un consentement verbal était suffisant pour démarrer l´étude.

**Traitement statistique:** les données ont été saisies et codées sur Excel. L´analyse statistique a été réalisée à l´aide du logiciel d´analyse statistique SPSS: *Statistical Package for Social Sciences (SPSS) for Windows, version 13.0 (SPSS, Inc, Chicago)*. L´analyse descriptive a consisté au calcul des fréquences absolues et relatives pour les variables qualitatives, et des paramètres de positionnement et de dispersion pour les variables quantitatives (moyenne, écart-type). L´étude de corrélation et la différence entre les groupes avec et sans TCA se sont fait par le test de student. Le seuil de significativité a été fixé à 5%. Ainsi, les résultats de corrélation sont considérés significatifs si p < 0,05.

## Résultats

**Les caractéristiques sociodémographiques:** sur un total de 550 questionnaires distribués, 506 ont été retenus pour analyse et seulement 44 questionnaires n´ont pas été retenus car ils n´avaient pas été bien ou totalement remplis, soit un taux de réponse à 92%. L´âge moyen des étudiants était de 22,17 ans avec des extrêmes allant de 18 à 29 ans. Nous avons une prédominance du sexe masculin (50,6%) par rapport au sexe féminin (49,4%). Le sexe ratio (H/F) était de 1,02. Le Tableau 1 représente une description des caractéristiques sociodémographiques de notre échantillon.

**Tableau 1:** caractéristiques sociodémographiques des participants à l’étude concernant les facteurs associés au risque de troubles du comportement alimentaire chez les étudiants en médecine de Casablanca (Maroc), durant l’année universitaire 2016-2017 (N = 506)

Paramètre	Pourcentage (%)
**Sexe**	
Hommes	50,6
Femmes	49,4
**Niveau d'études**	
1^e^ année	11
2^e^ année	14
3^e^ année	17
4^e^ année	22
5^e^ année	20
6^e^ année	16
**Niveau socio-économique**	
Bas	10,3
Moyen	78,7
Elevé	11
**Statut matrimonial**	
Marié	3,8
Célibataire	95,8
Divorcé	0,4
Veuf	0
**Nombre d'enfants**	
Sans	99
1-2	0,8
Plus de 3	0,2[CM1]

**Les caractéristiques cliniques:** au sein de notre échantillon, 2% des étudiants avaient des antécédents psychiatriques, et 7% présentaient un usage des substances psychoactives. Selon l´IMC, notre étude retrouvait 12,4% d´état de maigreur, 2,8% d´obésité et 14% de surpoids. Vingt-quatre virgule sept pourcent (24,7%) des étudiants avaient recours à des moyens de contrôle du poids (Tableau 2).

**Tableau 2:** caractéristiques cliniques des participants à l’étude concernant les facteurs associés au risque de troubles du comportement alimentaire chez les étudiants en médecine de Casablanca (Maroc), durant l’année universitaire 2016-2017 (N = 506)

Paramètre	Pourcentage (%)
**Antécédents psychiatriques**	2
**Usage des substances psycho-actives**	
Tabac	4,2
Alcool	1,6
Cannabis	1,2
**Répartition des étudiants selon l'indice de masse corporelle (IMC)**	
Maigreur	12,4
Corpulence normale	70,8
Surpoids	14
Obésité	2,8
**Répartition des étudiants selon les moyens de contrôle du poids**	
Régime alimentaire	10,1
Sport	9
Jeune	2
Laxatif	0,8
Coupe-faim	2
Vomissements provoqués	0,8

**Prévalence d'éventuels troubles du comportement alimentaire chez les étudiants en médecine:** le questionnaire SCOFF a détecté 127 personnes (25,09%) sur 506 susceptibles de souffrir de troubles du comportement alimentaire.

**Anxiété-dépression:** selon l´échelle HAD (Hospital anxiety and depression), 47,8% de la population étudiée présente des symptômes douteux d´anxiété et 8,3% une anxiété certaine. Alors que 14,4% des étudiants ont des symptômes douteux de dépression et 2% présentent une symptomatologie dépressive certaine.

**Evaluation du risque de TCA selon les facteurs associés:** parmi les facteurs que nous avons étudié, l´âge, le niveau d´études, l´utilisation des moyens de contrôle du poids, l´anxiété et la dépression sont des facteurs associés de façon significative au risque de présenter un trouble du comportement alimentaire (Tableau 3). D´autres facteurs ont été recherchés mais ils n´étaient pas significativement reliés à un risque de TCA tels que: sexe, état matrimonial, niveau socio-économique, consommation de substances et IMC.

**Tableau 3:** facteurs associés au risque de troubles du comportement alimentaire chez les étudiants de la faculté de médecine et de pharmacie de Casablanca (Maroc), durant l’année universitaire 2016-2017 (N = 506)

Paramètre	SCOFF positif (n=127)	SCOFF négatif (n=379)	Valeur de p
**Age (ans)**	21,39	22,43	<0,001
**Sexe**			
Hommes	63	194	0,459
Femmes	63	186	
**Niveau d'études**			
1^e^ année	28	27	<0,001
2^e^ année	29	41	
3^e^ année	11	77	
4^e^ année	28	80	
5^e^ année	15	88	
6^e^ année	15	66	
**Anxiété** (Selon l'échelle HAD)			
Pas de symptômes	51	130	0,007
Symptomatologie douteuse	61	207	
Symptomatologie certaine	15	42	
**Dépression** (Selon l'échelle HAD)			
Pas de symptômes	86	337	<0,001
Symptomatologie douteuse	31	31	
Symptomatologie certaine	9	1	
**Moyen de contrôle du poids**			
Régime alimentaire	4	1	0,004
Sport	22	28	0,001
Jeune	1	9	0,26
Laxatif	3	2	0,07
Coupe-faim	3	9	0,99
Vomissements provoqués	3	2	0,07

Abréviations: SCOFF: Sick, Control, One stone, Fat, Food HAD: Hospital anxiety and depression[CM1]

## Discussion

**Prévalence du risque de troubles du comportement alimentaire chez les étudiants en médecine:** concernant les méthodes d´évaluation, elles diffèrent d´une étude à l´autre ce qui rend les résultats très variables et difficiles à comparer. Un ensemble d´études utilisant le questionnaire SCOFF trouve des estimations de prévalence proches de 20% en population étudiante [13, 19, 20]. Nos résultats se rapprochent des données de la littérature. En effet, au sein de notre échantillon, 25,09% des étudiants sont susceptibles de souffrir de troubles du comportement alimentaire. Malgré les difficultés méthodologiques pour approcher la prévalence des TCA, la majorité des auteurs sont d´accord dans le sens d´une augmentation sensible de l´incidence actuelle des TCA chez les adolescents et les adultes jeunes [21, 22].

Une méta-analyse récente a apporté plus d´éclaircissement sur cette question. Selon les données de cette revue, La prévalence du risque de troubles du comportement alimentaire chez les étudiants en médecine était de 10,4%, avec des preuves statistiquement significatives d'hétérogénéité entre les études. Les estimations de prévalence entre les études variaient de 2,2 à 29,1% [23].

Des études transculturelles, mettant en évidence la montée d´incidence des troubles du comportement alimentaire dans des pays en voie de développement, ont fait pointer l´influence des médias à diffusion planétaire dans cette généralisation des troubles. Dans une perspective plus globale, ont été évoquées la mutation accélérée de l'identité et du rôle de la femme, ainsi que la quête de performance que la mondialisation provoquerait dans ces sociétés [24, 25]. Les taux de prévalences rapportées sont alarmants étant donné le fait que les troubles du comportement alimentaire ont le potentiel d'entraîner d'autres problèmes de santé physique ou mentale graves.

Des recherches antérieures montrent que plus de 80% des personnes à risque de TCA ont au moins un trouble psychiatrique comorbide, y compris des troubles anxieux (> 50%), des troubles affectifs (> 40%), et une consommation de substances (> 10%) [26, 27]. Les personnes atteintes de TCA peuvent développer des problèmes de santé physique, notamment des complications rhumatologiques [28], une insuffisance cardiaque [29], une pancréatite [30] et une infertilité [31]. Ainsi, il est clairement évident que les TCA ont des conséquences graves et persistantes sur la santé et par conséquent, l'identification et le traitement précoces sont importants.

### Facteurs associées au risque de troubles du comportement alimentaire chez les étudiants en médecine

**L´âge et le sexe:** les TCA ont été pour longtemps considérés comme une pathologie essentiellement féminine [32-34]. Selon les études, le risque de TCA chez les étudiants universitaires semble être lié au sexe [35, 36]: les TCA sont fréquents chez les étudiants particulièrement de sexe féminin. En effet, une étude menée au niveau de l´Université du Littoral Côte d´Opale sur 642 étudiants retrouve une suspicion de troubles du comportement alimentaire détectés en utilisant le SCOFF de l´ordre de 24,2% chez les sujets de sexe féminin versus 10,2% chez les sujets de sexe masculin. L´âge moyen est 22 ans [20]. Les résultats de la revue systématique de Jahrami *et al*. viennent appuyer ce constat, les étudiantes en médecine semblent plus à risque de développer un trouble du comportement alimentaire avec un taux de prévalence de 13,7%, l'âge moyen est de 21 ans (avec un intervalle de 18,5 à 25 ans) [23].

Dans notre étude, l´âge moyen est significativement associé au risque de TCA (l´âge moyen des étudiants était de 22,17 ans). En revanche, nous n´avons pas trouvé de différence significative entre les deux sexes (25,3% de sexe féminin versus 24,5% de sexe masculin). Les troubles de l'alimentation se manifestent principalement chez les jeunes femmes [37], du fait de la grande importance accordée à la minceur comme condition préalable à l'estime de soi [13, 27]. Néanmoins, des recherches récentes suggèrent que la prévalence et les manifestations des TCA chez les jeunes hommes augmentent dans les échantillons communautaires [38]. L'image corporelle masculine est apparue comme un facteur important d´augmentation de la prévalence des TCA chez les hommes [39].

**Le niveau d´étude:** certains chercheurs s´intéressent aux TCA en milieu étudiant, spécifiquement lors de la première année universitaire [20, 40]. L´incidence des TCA dans les premières années d´études ne cesse en effet d´augmenter au fil du temps [41]. C´est la tranche d´âge des 17-19 ans qui apparaît comme la plus critique, or, elle correspond à l´entrée dans les études supérieures [42]. Dans notre série l´étude analytique associant le niveau d´étude au risque de TCA a montré une nette augmentation de ce risque chez les étudiants surtout de la première et la deuxième année présentant ainsi les taux les plus significatifs. Les résultats de notre étude concordent avec les données de la littérature. La première année d´étude représente une période de transition avant la vie active et nécessite un ajustement important pour les étudiants. Les étudiants de première année doivent s´ajuster à deux types de transition, l´une développementale (de l´adolescence à l´âge adulte), l´autre éducationnelle (du lycée à l´université). Pendant la période transitoire que traversent ces jeunes apprenant peu à peu leur rôle d´étudiants, divers troubles ont été observés et notamment les TCA [43].

**Les moyens de contrôle du poids:** selon les études, le recours à des moyens pour contrôler le poids est fréquent chez les étudiants ayant des troubles du comportement alimentaire [44, 45]. Bennet *et al*. ont rapporté que les boulimiques se font vomir une à sept fois par mois [46]. En outre, une étude menée sur un échantillon de 1744 étudiants montre que parmi les facteurs de risque d´avoir une suspicion de TCA serait le fait d´avoir déjà fait un régime alimentaire [47]. En effet les résultats de notre étude vont dans le même sens, l´utilisation de moyens de contrôle du poids est reliée de façon significative au risque de TCA, et ce particulièrement pour le sport (p=0,001) et les régimes alimentaires (p=0,004).

**Anxiété, dépression, et risque de TCA:** les troubles du comportement alimentaire surviennent fréquemment avec d'autres troubles psychiatriques, tels que la dépression et les troubles anxieux [48]. Les études rapportent une forte comorbidité entre les TCA et les troubles anxio-dépressifs [26, 27]. Dans certaines études, près de 63% de patientes anorexiques présentent un trouble affectif sur la vie entière [49]. De même, 30 à 65% des anorexiques hospitalisées présentent des antécédents de troubles anxieux dont des troubles obsessionnels compulsifs (TOC) [50]. Bien que la cause exacte soit inconnue, on pense qu'une combinaison d'anomalies biologiques, psychologiques et/ou environnementales contribue au développement de TCA [51, 52]. La dépression, le stress et l'anxiété sont parmi les facteurs psychologiques associés aux troubles de l'alimentation [53].

En population étudiante, l´étude de Fragkos *et al*. menée sur un large échantillon de 1865 étudiants, trouve des corrélations significatives entre ces facteurs psychologiques et le risque de TCA. Ainsi la dépression, le stress et l´anxiété ont été retenus comme des facteurs de risque de troubles du comportement alimentaire chez les étudiants [36]. Les données de littérature confortent les résultats de notre étude, le risque de TCA a été significativement associé à l´augmentation du score d´anxiété (p=0,007) et de dépression (p<0,001) évaluées par l´échelle HAD. Lors de la transition vers l'âge adulte, les jeunes sont confrontés à de nombreux facteurs de stress pour devenir indépendants et commencer à se projeter dans l´avenir en termes de relations, de scolarité, de carrière, etc. [51, 54]. Les principaux facteurs de stress chez les étudiants universitaires sont les changements d'environnement, la charge de travail académique, les nouvelles relations et les problèmes de gestion du temps [55], ce qui les rend vulnérables aux troubles dépressifs, anxieux et aux TCA.

## Conclusion

Les étudiants constituent un groupe considéré comme à haut risque vis-à-vis des troubles du comportement alimentaire. Des particularités liées à leur travail (stress, irrégularité des horaires) et des facteurs socioculturels (idéal de minceur, autonomie par rapport à la famille) peuvent expliquer une telle vulnérabilité [42]. Les données de notre étude rejoignent celles de la littérature concernant la fréquence des troubles du comportement alimentaire en population étudiante et plus spécifiquement les étudiants en médecine. De même, des facteurs sociodémographiques, cliniques et psycho-pathologiques ont été associés au risque de TCA dans notre population.

En tant que futurs médecins, ces étudiants ont à répondre aussi bien pour eux-mêmes et pour leurs patients dans le cadre de leurs stages hospitaliers, à différentes questions concernant les TCA, mais également concernant les bonnes pratiques à adopter pour maintenir un poids stable tout en restant en bonne santé. Ainsi, mener des enquêtes de terrain en milieu universitaire prend toute son importance, non seulement pour pouvoir identifier les prévalences des troubles mentaux y compris les TCA chez les étudiants, mais surtout pour concevoir des stratégies efficaces et des interventions spécifiques de prise en charge et d´accompagnement psychiatrique et psychologique des personnes à risque. Dans ce sens, une revue systématique récente a révélé que les interventions psychologiques de prévention des troubles de l'alimentation en milieu universitaire peuvent avoir des effets importants, faibles à modérés sur les symptômes et les facteurs de risque des TCA. Un effet préventif significatif a été trouvé sur l'incidence des troubles de l'alimentation, suggérant une diminution de 38% de l'incidence dans les groupes d'intervention par rapport aux groupes témoins [56].

Compte tenu de l´impact négatives de longue durée des troubles de l'alimentation, de la forte prévalence des personnes d'âge universitaire à risque de développer un trouble de l'alimentation, et étant donné qu'environ 80% des personnes souffrant de TCA ne reçoivent pas de traitement [57, 58], la prévention précoce de ces troubles devrait être une priorité de santé publique. Les universités pourraient être un cadre optimal pour les interventions préventives contre les troubles de l'alimentation, étant donné que les universités peuvent généralement fournir l'infrastructure à grande échelle nécessaire pour recruter, diffuser et offrir de telles interventions, sur place ou via Internet [56] .

Notre travail permet de donner un aperçu sur l´importance de la prévalence des TCA dans une population faite de futurs médecins, et de poser certains questionnements quant au degré de conscience et de connaissance de ces pathologies répandues en milieu universitaire. Néanmoins, nos résultats nous amènent à être prudents quant à la lecture et l´interprétation des données recueillies. En effet, parmi les limites de cette étude, son caractère transversal, le choix de la population, le volontariat qui peut écarter des sujets qui peuvent avoir une approche de fuite devant l´évaluation comme l´ont observé Kim *et al*. [59]. La reproduction de cette étude dans le cadre d´un suivi de la cohorte des étudiants pourra, à l´avenir, pallier certains de ces biais. En outre, mener d´autres études sur une population plus variée pourraient également être envisagées afin d´avoir une vision plus globale de la situation au Maroc.

### Etat des connaissances sur le sujet

- Les étudiants constituent un groupe considéré comme à haut risque vis-à-vis des troubles du comportement alimentaire (TCA);
- Malgré les difficultés méthodologiques pour approcher la prévalence des TCA, la majorité des auteurs sont d´accord dans le sens d´une augmentation sensible de l´incidence actuelle des TCA chez les adolescents et les adultes jeunes;
- Les TCA sont d´origine multifactorielle et sont imputables à une association de facteurs de vulnérabilité biologique, sociale et psychologique.

### Contribution de notre étude à la connaissance

- Les données de notre étude rejoignent celles de la littérature concernant la fréquence des troubles du comportement alimentaire en population étudiante et plus spécifiquement les étudiants en médecine;
- Dans notre étude, l´âge, le niveau d´études, l´utilisation des moyens de contrôle du poids, l´anxiété et la dépression sont des facteurs associés de façon significative au risque de présenter un trouble du comportement alimentaire;
- La littérature occidentale concernant le risque de TCA en population étudiante est plus abondante par rapport à celle des pays d´Afrique. Ainsi, les résultats de notre étude contribuent à avoir une vision sur la situation au Maroc et encouragent à mener d´autres études plus avancées méthodologiquement et sur des populations plus variées.
